# A screen of *Salmonella enterica* mutants interacting with fresh onions and alfalfa sprouts

**DOI:** 10.1016/j.ijfoodmicro.2026.111696

**Published:** 2026-03-02

**Authors:** Laura Führer, Steffen Porwollik, Weiping Chu, Verena Hohenester, Irmak Sah, Michael McClelland, Claudia Guldimann, Irene Esteban-Cuesta

**Affiliations:** a Competence Center for Food Safety, Chair for Food Safety and Analytics, Veterinary Faculty, LMU Munich, Oberschleissheim, Germany; b Department of Microbiology and Molecular Genetics, School of Medicine, University of California, Irvine, CA, United States of America

**Keywords:** Transposon insertion sequencing, Food safety, Foodborne pathogen, Ready-to-eat products, Salmonellosis, Microbial contamination, Fresh produce

## Abstract

*Salmonella enterica* is a zoonotic pathogen that primarily causes disease through the contamination of food products. Foodborne salmonellosis outbreaks have frequently been associated with the consumption of contaminated ready-to-eat foods, including sprouts and fresh diced onions. To better control *Salmonella* throughout the food chain and enhance food safety, it is crucial to understand the interaction mechanisms of *S. enterica* with relevant food matrices. To identify the genetic determinants underlying the persistence of *Salmonella* on sprouts and onions, we analyzed barcoded transposon mutant libraries in two relevant *S. enterica* serovars, Typhimurium and Enteritidis, on these two food matrices for five days under refrigerated storage conditions. Key fitness determinants of *S. enterica* were observed to be under selection, including virulence factors, two-component systems, flagellar assembly, cell wall integrity, RNA degradation, and mismatch repair systems. The phenotype of selected *S*. Typhimurium mutants was subsequently verified in competition assays. Our study showed that disruption of *tolC*, encoding a multidrug efflux pump, resulted in a greater fitness reduction on onions than on sprouts and in nutrient-rich media. Furthermore, the fitness disadvantage of mutants in *oxyR*, a key regulator of the oxidative stress response, was much reduced on sprouts, compared to onions or standard growth conditions. This study provides a comprehensive overview of the genetic determinants that influence the interaction of *S. enterica* with these fresh produce items under real-world conditions.

## 1. Introduction

*Salmonella enterica* subsp. *enterica* (*S. enterica*) is one of the most important foodborne pathogens worldwide. Salmonellosis was the second most reported zoonosis in 2023, causing 77,486 human cases in the EU (EFSA and ECDC, 2024). *Salmonella* foodborne outbreaks have frequently been associated with contaminated fresh produce and ready-to-eat (RTE) food items (Ma et al., 2024; Olaimat and Holley, 2012). These products pose a higher risk to consumers, because they are intended for raw consumption without prior pathogen inactivation.

Fresh onions (*Allium cepa* L.) are often sold diced, as ready-to-eat (RTE) products, or included in mixed food products sold “to go” (e.g., fresh salads, kebabs, wraps). Although RTE onions are minimally processed, microbial contamination may occur at different stages of the production process. These stages include preselection, peeling, cleaning, cutting, packaging, and storage (Bahram-Parvar and Lim, 2018; Balali et al., 2020; EFSA BIOHAZ Panel, 2014; Savitha et al., 2022). Diced onions are often stored under refrigeration at serving counters, which could pose an additional risk of contamination during serving and handling. Onions may have a reduced risk of microbial contamination due to their inherent natural antimicrobial compounds (Kyung, 2012; Lanzotti et al., 2013; Santas et al., 2010; Sharma et al., 2018) and a relatively low pH of approximately 5.5 at 10 °C, which may further decrease during storage (Berno et al., 2014). Their natural microbiota consists primarily of spoilage microorganisms, such as bacteria, yeasts, and molds. After dicing, the microorganism counts usually range from 2.0 to 4.0 log_10_ CFU/g, and may increase during storage (Bahram-Parvar and Lim, 2018; Blanchard et al., 1996; Liu and Li, 2006). Despite this challenging environmental conditions, *S. enterica* is known to be able to persist on this food matrix (Lieberman et al., 2015; Yemmireddy and Mancias, 2025) and has been recently the cause of several foodborne outbreaks due to the contamination of raw onions in the US and Canada between 2020 and 2023 (Denich et al., 2024; Mitchell et al., 2024).

Sprouted seeds have also been source of several salmonellosis outbreaks in recent years. Sprouts are food products obtained from the germination of seeds, followed by their subsequent development in water, and harvested before true leaves form (Official Journal of the European Union, 2013). In 2023, sprouts were identified as the food item with the highest *S. enterica* prevalence among all tested RTE food samplings in the EU (EFSA and ECDC, 2024). Specifically, alfalfa sprouts (*Medicago sativa* L.) have been associated with salmonellosis outbreaks since 1994, with the latest outbreak continuing into 2025 in the EU (EFSA Panel on Biological Hazards (BIOHAZ), 2011; Ma et al., 2024; Rakover et al., 2025). The warm and humid conditions required for sprout production provide an optimal environment for bacterial growth and contamination with *S. enterica* or other enteric pathogens can occur at nearly every production stage (EFSA and ECDC, 2024; BIOHAZ, 2011; NACMCF, 1999). This is also reflected in its diverse background microbiota, mainly including *Enterobacteriaceae* and *Pseudomonadaceae*, and mesophilic aerobic populations on the finished product that may reach 10^9^ CFU/g (Abadias et al., 2008; Jang et al., 2021; Keshri et al., 2019; Kim et al., 2013; Viswanathan and Kaur, 2001; Waje et al., 2009; Young Kim et al., 2022). Hygienic measures such as seed treatment and control of irrigation water may reduce the bacterial load on sprouts but do not fully eliminate pathogens (Ding et al., 2013; Kim et al., 2009; Sikin et al., 2013), making sprouts a challenging food product in terms of food safety.

Quantitative data provide insight into the population dynamics of different microorganisms on the food matrix under specific conditions of interest. However, it remains crucial to understand the genetic determinants and metabolic pathways underlying the resilience of *S. enterica* on relevant food matrices, to enhance food safety and inform the future development of highly targeted preventive measures.

During growth on the food matrix, *S. enterica* encounters various stress conditions, including low temperatures and an acidic environment. There is a growing understanding of the mechanisms behind the general stress response of *S. enterica* (Pradhan and Devi Negi, 2019; Spector and Kenyon, 2012), with an increasing number of studies that have directly investigated the interaction mechanisms of *S. enterica* with relevant food matrices, including tomatoes (de Moraes et al., 2017; Noel et al., 2010), low-water activity foods (de Moraes et al., 2017; Jayeola et al., 2020; Li et al., 2020) and RTE muskmelons (Esteban-Cuesta et al., 2026). These studies have identified interaction mechanisms necessary for the survival of *S. enterica* on the food matrix, which include cell wall integrity, lipopolysaccharide (LPS) biosynthesis, and motility, as well as mechanisms related to general growth, such as DNA repair, and the biosynthesis of amino acids and nucleotides. However, each food matrix presents a unique environment that may require different microbial fitness mechanisms.

No studies have been conducted to date to evaluate the genetic determinants of *Salmonella* survival on fresh diced onions. However, the colonization and attachment mechanisms of *S. enterica* during the germination and sprouting of alfalfa have been investigated in comparison to attachment on glass surfaces (Holden et al., 2024). The study identified type III secretion systems as key mechanisms for early-stage colonization, and DNA housekeeping and envelope synthesis in subsequent colonization phases. During later interaction stages, LPS and flagellar synthesis and the global stress response played essential roles. However, the mechanisms necessary for the bacterium’s survival during cold storage in supermarkets and households have not yet been assessed.

We hypothesized that distinct genetic mechanisms influence the resilience of *S. enterica* on fresh diced onions and alfalfa sprouts and that these mechanisms may differ between serovars. Therefore, two barcoded transposon libraries, one in *S. enterica* serovar Enteritidis and one in serovar Typhimurium, were analyzed on both food matrices under abusive cold storage conditions at 8 °C, temperature frequently encountered in open cooling displays or household refrigerators (Chaomuang et al., 2017; Evans and Redmond, 2015; Laguerre et al., 2002).

## 2. Materials and methods

### 2.1. Bacterial strains and library construction

*S*. Enteritidis PT4 P125109 (Toro et al., 2016) (SEN, NCBI accession no. CP063700.1/CP063701.1), a clinical strain associated with a poultry outbreak in the United Kingdom, and *S*. Ttyphimurium ATCC 14028, isolated from a 4-week-old chicken in 1960 (Jarvik et al., 2010) (STM, NCBI accession no. CP001363.1/ CP001362.2), were used to create the two barcoded transposon mutant libraries employed in this study. The wild-type strains were preserved at −80 °C in Luria-Bertani broth (LB; Carl Roth GmbH & Co. KG, Karlsruhe, Germany) with a final concentration of 20% glycerol (Th. Geyer GmbH & Co. KG, Renningen, Germany) and cultured in LB broth or 1.5% LB agar (Oxoid Deutschland GmbH, Wesel, Germany).

Library construction, mapping of the barcoded transposons, and annotation of the library were previously described in de Moraes et al. (2017) and Li et al. (2020). Briefly, a Tn*5* derivative with an N_18_ random barcode was introduced in each genome using the EZ-Tn*5* 〈T7/KAN-2〉 promoter insertion kit (Epicentre Biotechnologies, Madison, WI, United States). The transformed cells were collected after overnight culture at 37 °C on LB agar supplemented with 60 μg/ml kanamycin (LB^Kan^) final concentration. The resulting libraries had a complexity of approximately 230,000 mutants for *S*. Typhimurium ATCC 14028 (de Moraes et al., 2017) and 140,000 mutants for *S*. Enteritidis PT4 P125109 (Li et al., 2020).

### 2.2. Comparative growth analysis

Growth analysis of the *S. enterica* barcoded transposon mutant libraries on alfalfa sprouts and fresh diced onions was performed as described in Esteban-Cuesta et al. (2026). Inoculation was performed as described in the library screening procedure below, and populations were enumerated 1 h after inoculation (d_1_) and every 24 h for five days (d_1_-d_5_). Five biological replicates were performed, and the results were analyzed using an ANOVA test. A *p*-value ≤ 0.05 was considered significant.

### 2.3. Screenings of S. enterica TIS libraries on fresh diced onions and alfalfa sprouts

TIS library screenings on alfalfa sprouts and fresh-diced onions were performed mainly as described by Esteban-Cuesta et al. (2026). Briefly, library stocks were thawed, and 300 μl were propagated in 30 ml LB^Kan^ (60 μg/ml, Carl Roth) broth at 37 °C and 200 rpm until the early stationary phase was reached, which corresponds to OD_600_ 1. 1 ml of the culture was diluted 1:10 in 9 ml PBS and centrifuged at 4500 rcf for 5 min, the supernatant was discarded, and the inoculum was prepared by resuspending the cell pellet in phosphate-buffered saline (PBS; Carl Roth) at a 1:5 (pellet: PBS) volume ratio.

Onions (*Allium cepa* L.) and alfalfa sprouts (*Medicago sativa* L.) samples were bought at local grocery stores. The background microbiota of the uninoculated food matrices was analyzed by assessing the mesophilic aerobic bacteria (MAB; EN ISO 4833–2:2013) and *Enterobacteriaceae* (EN ISO 21528–2:2017; anaerobic incubation: O_2_ < 0.1%, CO_2_ 7.0–15.0%) at each sampling time point. The absence of prior *Salmonella* spp. on the food matrices was ensured by qualitative microbiological analysis according to EN ISO 6579–1:2017, A1:2020.

Onions were peeled and diced with sterile knives. 10 g of food matrix was weighed into sterile blender bags (Size 400 ml, Avantor VWR International GmbH, Darmstadt, Germany) and incubated with 5 ml of the inoculum. The final concentration was approximately 7.0 log_10_ CFU/g, to ensure that the complexity of the library was maintained. The inoculum was evenly distributed to ensure complete coverage of the food matrix. *No matrix* samples consisting of the inoculum without a food matrix in 5 ml PBS in sterile blender bags were included as negative controls.

Inoculated samples were incubated at 8 °C for a total of 96 h (4 days), with samples taken from the inoculum (I), after 1 h (d_1_), 48 h (d_3_), and 96 h (d_5_) post inoculation. Samples were transferred into 90 ml LB^Kan^ broth and incubated at 37 °C and 200 rpm for 30 min to enable detachment of the bacteria from the food matrix. *S. enterica* mutant populations were enumerated after serial dilutions in PBS. To remove food matrix residues, samples were subsequently filtered and centrifuged at 4500 rcf for 5 min. The supernatant was discarded, and the pellet resuspended in 90 ml LB^Kan^. The washed cells were incubated at 37 °C and 200 rpm for 7.5 h and then stored at −80 °C for subsequent analysis. Given the importance of experimental consistency, five biological replicates were performed to ensure the robustness and reliability of the results.

Sequencing library preparation, Illumina sequencing, and raw read processing were performed as previously described in Esteban-Cuesta et al. (2026). For library preparation, the frozen samples (40 μl) were washed three times in water. The washed cells were digested with 1.5 μl proteinase K (concentration 100 mg/ml, recombinant proteinase K (AppliChem GmbH, Darmstadt, Germany)) in 15 μl 2x lysis buffer (20 mM Tris [pH 8.0], 2 mM EDTA, 0.2% Triton X-100; Carl Roth) for 2 h at 55 °C. The transposon insertion sites were amplified via PCR using a reaction mixture with 25 μl Q5^™^ Hot Start High-Fidelity, 2x Master Mix (New England Biolabs Inc., Ipswich, USA) and 5 μl DNA template. L and V Primers (Esteban-Cuesta et al., 2026) containing a transposon-specific sequence and custom Illumina adapters were used at a final concentration of 0.2 μM.

5 μl of the successful PCR products were pooled and purified using the QIAquick PCR Purification Kit (Qiagen, Maryland, USA) according to the manufacturer’s instructions. Followed by Illumina-sequencing using a NovaSeq 6000 on a dual-indexed paired-end 100 or 150 base run with one million reads per sample. Raw sequencing reads were demultiplexed according to their indexes and processed using in-house Python scripts to detect and quantify barcodes flanked by the expected genomic sequence.

Since multiple transposon insertions can map to the same gene and each is associated with a distinct barcode, the barcode insertions were compiled into aggregated counts for each disrupted gene. Differences in the aggregated mutant abundances between time points as well as between food samples and negative controls, were statistically analyzed using DESeq2 (Love et al., 2014) and the log_2_ fold changes (fc) were reported. Mutations were considered to have a significant fitness effect for *S. enterica* if they fulfilled a dual requirement for at least one sampling time point: (i) a log_2_ fc > |1.0| with a *p*_adj_-value ≤ 0.01 in the comparison between food samples and *no matrix* samples in PBS, and (ii) fulfilled the same statistical threshold (log_2_-fc > |1.0|, *p*_adj_ ≤ 0.01) when comparing the inoculum to food sample at the same sampling time point. For the final sampling time point, d_5_, criterion (ii) was also fulfilled in comparisons between the first and the final food sampling time points (d_1_ vs d_5_). This strict criterion was implemented to identify the genes with the most significant role during the interaction with the food matrix.

For KEGG and GO enrichment analyses (Ashburner et al., 2000; Gene Ontology Consortium, 2023; Kanehisa et al., 2023; Thomas et al., 2022) the threshold for significance was relaxed to *p*_adj_ < 0.05. KEGG enrichment analysis was carried out using enrichKEGG from ClusterProfiler (Wu et al., 2021) and GO enrichment analysis used TopGO (Alexa and Rahnenfuhrer, 2024) in R (v.4.4.1). KEGG pathways with a *q*-value ≤ 0.01 and GO terms with a *classic fisher* ≤ 0.01 were considered to be significantly enriched.

### 2.4. Competition assays

Candidate genes identified in the TIS analysis were subjected to direct competition between available *S*. Typhimurium 14028 single-gene deletion mutants (SGDs) and the *S*. Typhimurium 14028 wild-type strain. The *S*. Typhimurium 14028 SGD collection (Porwollik et al., 2014) had two mutants available for most genes: one mutant harboring a kanamycin resistance gene in the sense direction of the deleted gene (SGD^kan^) and one mutant harboring a chloramphenicol resistance gene in the antisense direction (SGD^cm^). The wild-type strain (*S*. Typhimurium 14208s) used for these competition experiments was isogenic except for a tetracycline resistance inserted in *malXY* (WT_STM_Tet^R^). The individual mutants were stored at −80 °C in LB-broth with a final concentration of 20% glycerol and supplemented with a final concentration of 60 μg/ml kanamycin (LB^Kan^), 20 μg/ml chloramphenicol (LB^Cm^) or 15 μg/ml tetracycline (LB^Tet^, all Carl Roth).

The selection of candidate genes was based on the fitness effects observed in the TIS data and whether the SGD was available in the collection. Competition assays were performed as described in Esteban-Cuesta et al., 2026. In short, a mixture of 1:1:1 (SGD^kan^:SGD^cm^: WT_STM_Tet^R^) was inoculated at a concentration of 10^7^ CFU/g or ml to the food matrix and stored at 8 °C for 5 days. To confirm the phenotype of the selected mutants, competition indices were calculated by comparing colony count changes of the respective sense and antisense mutants with those of the wild-type strain WT_STM_Tet^R^. A negative effect was deemed confirmed if the average competitive index $\left(\frac{\left(\frac{\text{mutant}}{\text{wildtype}}\right)\text{timepoint}}{\left(\frac{\text{mutant}}{\text{wildtype}}\right)\text{inoculum}}\right)$ of each experiment plus one standard deviation was <1. If the average competitive index minus one standard deviation was >1, a positive fitness effect was considered confirmed. Competition assays were performed in triplicate, except for those assays where no fitness effect was observed in two replicates, after which the assay was discontinued.

## 3. Results

### 3.1. Growth analyses

#### 3.1.1. Dynamics of the Transposon Insertion Sequencing (TIS) libraries on food matrices

No statistically significant differences were observed between the population dynamics of the *S. enterica* barcoded transposon libraries and their corresponding wild-type strains on alfalfa sprouts and fresh diced onions, except for SEN on onions at d_3_, where the library counts were significantly higher (0.4 log_10_ CFU/g, *p* = 0.014) than those of the wild-type (Fig. S1). After 96 h of incubation at 8 °C (d_5_), populations of the STM and the SEN libraries decreased on alfalfa sprouts by 0.2 and 0.3 log_10_ CFU/g, respectively, compared to the inoculum. On fresh diced onions, counts of the STM library decreased by 0.3 log_10_ CFU/g, while for the SEN library, a reduction of 0.2 log_10_ CFU/g was observed.

During the screenings the STM library displayed significantly higher CFU counts (*p* = 0.03) than its SEN counterpart at d_5_ on onions (Fig. S2). Significantly higher population were observed on alfalfa sprouts compared to fresh diced onions for both the STM (d_1_ *p* = 0.03) and the SEN library (d_1_ *p* = 0.01; d_3_ *p* = 0.03).

#### 3.1.2. Background microbiota

During cold storage, mesophilic aerobic bacteria (MAB) counts on onions increased from approximately 3.0 to 6.8 log_10_ CFU/g while *Enterobacteriaceae* populations increased from 0.8 to approximately 2.0 log_10_ CFU/g. On alfalfa sprouts, both MAB and *Enterobacteriaceae* were detected at higher levels than on onions. After 96 h of storage at 8 °C, MAB populations increased slightly from 8.7 log_10_ CFU/g to 9.0 log_10_ CFU/g, and *Enterobacteriaceae* remained stable at approximately 7.8 log_10_ CFU/g (Fig. S3).

### 3.2. TIS data analysis: genes under selection on fresh diced onions and alfalfa sprouts

Notable differences were observed between the TIS analysis results for the interaction of *S. enterica* with fresh diced onions and alfalfa sprouts, as well as between the two *S. enterica* TIS libraries.

**Fig. 1.**
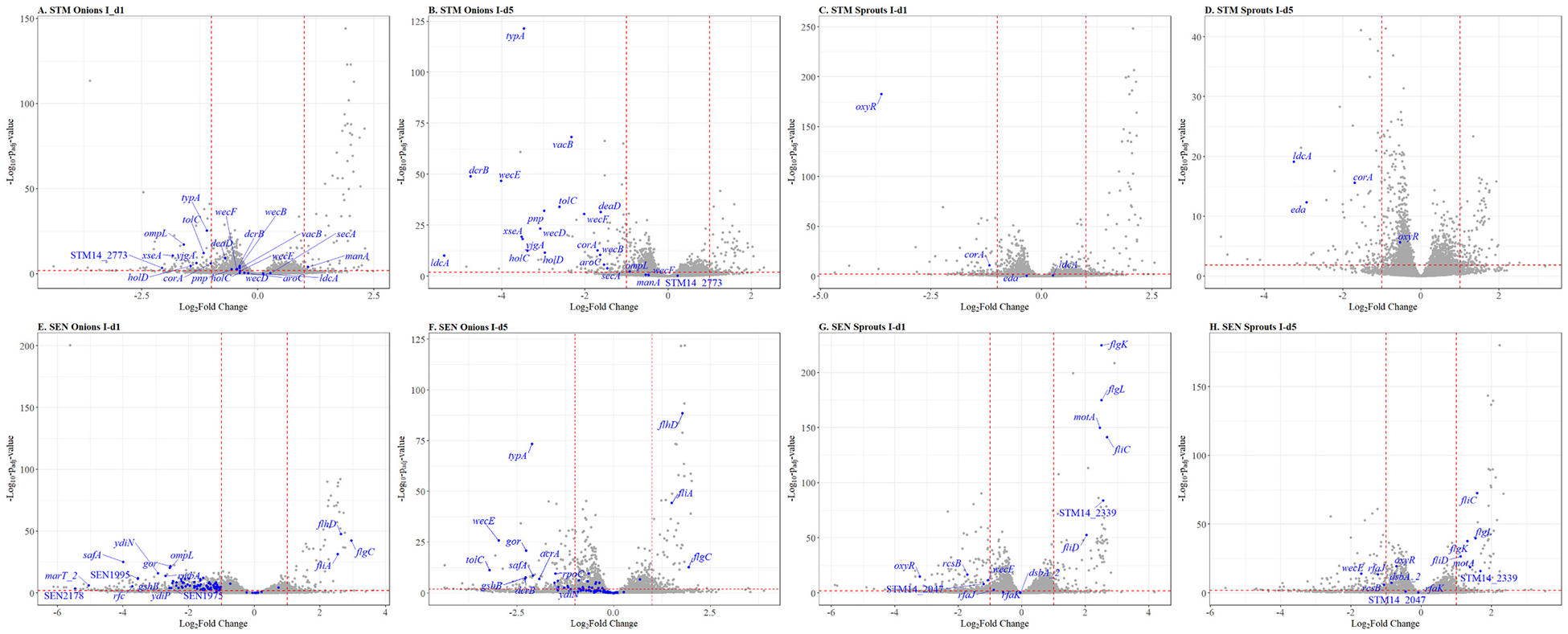
Changes in the aggregated mutant abundances of the barcoded transposon mutant libraries during interaction with fresh diced onions and alfalfa sprouts at 8 °C. Volcano plots exhibit fitness effects for the library mutants with the aggregate mutant abundances on the X-axis and the corresponding *p*-value on the Y-axis. Panels A–D correspond to the STM library on both onions (A, B) and alfalfa sprouts (C, D). Panels E–H correspond to the SEN library (E, F: onions; G, H: alfalfa sprouts). Panels A, C, E and G correspond to the comparison between inoculum and 1 h after incubation (I-d_1_), while panels B, D, F, and H depict comparison between the inoculum and end of storage (96 h, I-d_5_). Dots represent the aggregate mutant abundances for one specific gene. Mutations in genes with a significant fitness effect are shown in blue and selected genes were labeled. Insertions in intergenic regions with significant fitness effects were excluded.

For the interaction of SEN on fresh diced onions, transposon insertions in a total of 193 genetic elements caused a significant fitness effect, including 44 insertions located in intergenic regions (IR), one in a pseudogene, and 56 in genes encoding hypothetical proteins (HPs), of which 41 had putative functions assigned (Table 1). Most of these mutants displayed a negative fitness effect, except for three mutants, *fliA*, *flhD*, and *flgC*, which were positively selected within the first hour of interaction and maintained a fitness advantage throughout the entire screening (Fig. 1 and Fig. S4). Insertions in numerous genes resulted in a negative selection within the first hour of storage, which subsequently diminished over the remaining storage period.

The interaction of the STM library with onions resulted in considerably fewer genes (*n* = 21) with a significant fitness effect, where all but *STM14_2773* were negatively selected (Table 1, Fig. 1 and Fig. S4).

On alfalfa sprouts, 13 genes with a fitness effect were observed for the SEN library; six elements conferred a positive fitness effect when disrupted by transposons, while seven genes were negatively selected (Table 2, Fig. 1 and Fig. S4). Disruption of 5 genetic elements resulted in a significant fitness effect for STM (Table 2, Fig. 1 and Fig. S4), including one in an IR. Mutants in *oxyR* exhibited a dynamic behavior that shifted from being negatively to positively selected, whereas all others were negatively selected.

The TIS approach revealed common interaction mechanisms between the two serovars and between the two food matrices. On fresh diced onions, disruption of *dcrB*, *ompL*, *tolC*, *typA*, and *wecE* resulted in a significant fitness effect for both *S. enterica* serovars. On alfalfa sprouts, o*xyR* was commonly identified in STM and SEN. The *rcsB* and *wecE* genes were relevant for SEN on both food matrices, while STM relied on *corA* and *ldcA* on both food matrices. The complete results of the TIS analysis are presented in Table S1.

Significantly enriched KEGG pathways (*q-value* ≤ 0.01) and associated GO terms (*classic Fisher* ≤ 0.01) are summarized in Table 3, with full results in Table S2.

In summary, two-component systems (TCSs), T3SS, efflux pumps, flagella, ribosome biogenesis, and LPS biosynthesis were revealed as important mechanisms for the interaction of SEN with fresh diced onions On alfalfa sprouts, TCSs, bacterial chemotaxis, and the response to ROS (reactive oxygen species) played a crucial role. The main interaction mechanisms identified for STM were genetic information processing and modification of the cell envelope.

### 3.3. Competition assays

A collection of genes displaying a significant fitness effect on the respective food matrix was selected for competition assays. A total of 15 genes for fresh diced onions and four for alfalfa sprouts were selected from the single-gene deletions mutant collection in *S*. Typhimurium 14028 and subjected to competition assays on the food matrix, in LB, and in PBS (Table 4). From these, three genes - *corA*, *ldcA* and *xseA* - were tested on both food matrices. Additionally, *wecC* was selected for competition assays on onions given the overrepresentation of the *wec*-genes in the TIS data and KEGG analysis. Due to the strong negative effect of *xseA* on onions and melons (Esteban-Cuesta et al., 2026), competitions on *xseA* were expanded to sprouts. Although the threshold for significance was not fulfilled on this food matrix. On diced onions, the fitness effect observed in the TIS assays was confirmed for 10 of the 15 genes tested. On alfalfa sprouts, the fitness impact of all four tested genes was confirmed. However, most of the tested mutants also had a fitness effect in nutrient-rich media. *tolC* showed a considerably stronger negative fitness effect on fresh-diced onions compared to LB. In contrast, o*xyR* showed a positive polar effect on sprouts and a negative fitness effect in LB. Additionally, *deaD* and *vacB* displayed fitness effects in LB that were absent in our competition assays in onions. In PBS, two genes, *corA*, and *xseA*, showed polar effects, while *ldcA* also displayed a fitness effect under these nutrient-restricted conditions.

## 4. Discussion

Fitness determinants for the interaction of *S. enterica* on fresh diced onions and alfalfa sprouts were investigated using barcoded transposon libraries in *S*. Enteritidis P125109 (SEN) and *S*. Typhimurium 14028 (STM).

The food matrices analyzed in this study, fresh diced onions and alfalfa sprouts, provide two distinct environmental conditions for *S. enterica*. This is reflected in the TIS results, where screenings of the *S. enterica* libraries yielded notably fewer mutants with a fitness effect on alfalfa sprouts than on onions. Key differences between these food matrices include their inherent pH as well as their composition. Onions provide a more challenging acidic environment with a pH ranging from 5.3 to 5.9 (Berno et al., 2014; Roldán-Marín et al., 2009). Although the pH of alfalfa sprouts has not been comprehensively characterized, they are commonly assumed to represent a less acidic environment compared with onions, which may contribute to the observed differences. Furthermore, onions are known to produce natural antimicrobial substances (Al-Delaimy and Ali, 1970; Kyung, 2012; Lanzotti et al., 2013; Santas et al., 2010; Sharma et al., 2018; Zohri et al., 1995), while alfalfa sprouts also produce bioactive compounds, such as specific vitamins and antioxidants (Benincasa et al., 2019). The majority of SEN mutations with fitness effects were under strong negative selection in onions within the first hour of interaction, which then switched to positive selection for the remainder of the storage period. Many of these mechanisms may therefore only be required for the initial adaptation to intrinsic stress factors present in onions and allow subsequent cell growth.

Alfalfa sprouts supported the maintenance of both libraries significantly better than fresh diced onions in the first hour of interaction. The growth potential for both *S. enterica* strains was comparable to previous studies with *S. enterica* wild-type strains on onions (Lieberman et al., 2015; Yemmireddy and Mancias, 2025) and sprouts (Charkowski et al., 2002; Kim et al., 2018). Minimal variations could be attributed to different time-temperature combinations or inoculum concentrations.

Alfalfa sprouts and fresh diced onions also differ in their initial background microbiota. While alfalfa sprouts had high MAB and *Enterobacteriaceae* populations of about 9.0 and 8.0 log_10_ CFU/g, respectively, lower counts were found on onions, where MAB were at 3.0 log_10_ CFU/g and *Enterobacteriaceae* at 0.8 log_10_ CFU/g. These results correlate well with previous studies (Abadias et al., 2008; Iacumin and Comi, 2019; Jang et al., 2021; Keshri et al., 2019). However, after five days, while counts on alfalfa sprouts remained stable, the MAB increased to almost 7.0 log_10_ in the onion samples.

Strain-specific differences were observed during the TIS experiments. In general, the SEN library maintained lower CFUs than the STM library on onions, and substantially more SEN insertion mutants displayed a significant fitness effect. This higher sensitivity of SEN under the stress conditions posed by these food matrices of plant origin may represent strain- or serovar-specific effects. We note that salmonellosis outbreaks caused by SEN strains are mostly associated with poultry and poultry-derived products, including eggs and egg products, but are rarely associated with foods of plant origin (EFSA and ECDC, 2024). In contrast, *S*. Typhimurium strains have been associated with a broader food spectrum that ranges from pork or beef meet (EFSA and ECDC, 2024) to contaminated chocolate (EFSA and ECDC, 2023) and fresh produce (Colombe et al., 2019; Seelman Federman et al., 2024). A recent study also demonstrated that *S*. Typhimurium exhibits higher resilience to stress conditions in the food chain compared to other *S. enterica* strains (Pye et al., 2023).

STM relies on mechanisms such as RNA and DNA degradation (*deaD*, *pnp*, *vacB*), mismatch repair (*holCD*, *xseA*), and ribosome biogenesis *(yjgA*, *typA*) to sustain itself on these food matrices, which are baseline mechanisms for any cell growth. Previous studies also identified DNA repair to be relevant for *S. enterica* on low moisture foods, alfalfa sprouts, and muskmelons (Brankatschk et al., 2014; Esteban-Cuesta et al., 2026; Li et al., 2020). Collectively, these mechanisms enable the cell to utilize nutrients and metabolites more efficiently, which is also exemplified by the fitness effect observed in *eda* and *aroC* STM mutants. The 2-keto-3-deoxy-6-phosphogluconate aldolase encoded by *eda* is involved in the Entner-Doudoroff pathway, a more energy-efficient alternative to glycolysis that may be more metabolically favorable under different environmental conditions, such as carbon and phosphate starvation (Gupta and Gupta, 2021; Murray and Conway, 2005). AroC is a chorismate synthase that catalyzes the last step in the synthesis of chorismate within the shikimate pathway. This pathway is responsible for the de novo biosynthesis of aromatic amino acids, rendering the cell prototrophic for aromatic amino acids and other nutrients for which chorismate serves as a precursor (Bentley and Haslam, 1990; Charles et al., 1990; Dosselaere and Vanderleyden, 2001). Taken together, the fitness effects of *eda* and *aroC*, along with those related to RNA degradation, mismatch repair, and tRNA and ribosome biogenesis, suggest that STM may struggle to scavenge certain nutrients from the food matrix and heavily relies on its own metabolic resources.

KEGG enrichment analysis revealed both positively and negatively selected genes within the same system category. This reflects the functional diversity within the systems, where individual genes act as activators or repressors depending on environmental conditions, or are parallel systems with different functions, such as two-component systems. Mutations in two-component systems (TCSs) were detrimental to the maintenance of SEN and STM on both onions and alfalfa sprouts at 8 °C. TCSs are signal-transduction systems composed of a sensor kinase and a response regulator that enable bacteria to sense and respond to changes in their environment (Beier and Gross, 2006). The fitness effect observed for several SEN mutants with insertions in TCS-related genes on onions (*rcsB*, *ompC*, *fimZ*, *phoN*, *wecB*, *ssrA*, *fliA*, *flhD*, *sthAB*, *tolC*) and sprouts (*rcsB*, *fliC*, *motA*, *wecE*) is also supported by the enrichment of the corresponding KEGG pathway for both matrices, while TCS-related genes were also relevant for the interaction of STM with onions (*ompL*, *tolC*, *wecBDEF*). TCSs are involved in a wide variety of mechanisms. The EnvZ-OmpR TCS, crucial for SEN on onions, is primarily related to osmotic stress. However, it has an additional role in the acid stress response (Chakraborty et al., 2015; Chakraborty and Kenney, 2018), making it important for the interaction with onions (Bernardini et al., 1990; Sleator and Hill, 2002; Tipton and Rather, 2017; Xiao et al., 2022). Additionally, the EnvZ-OmpR TCS affects the expression of virulence factors, such as flagellar assembly and type III secretion systems (T3SS), in response to low pH (Kenney and Anand, 2020; Shin and Park, 1995). The EnvZ-OmpR TCS was previously found to be relevant for the survival of *S*. Newport on muskmelons (Esteban-Cuesta et al., 2026) and under desiccation stress on pistachios (Jayeola et al., 2020).

Modifications of the cell envelope are important mechanisms bacteria use to adapt to different environmental stressors. Genes related to cell envelope functions were commonly identified for both *S. enterica* serovars on fresh diced onions and alfalfa sprouts. For instance, STM *ldcA* mutants were negatively selected on both food matrices. *ldcA* encodes for the L,D-carboxypeptidase LdcA that plays a key role in the peptidoglycan recycling pathway during cell wall synthesis. This phenotype was confirmed in competition against the wild-type on the food matrix, in LB, and PBS, indicating that *ldcA* mutants are generally growth-impaired. LPS biosynthesis was also found to be relevant for the survival of *S. enterica* on low-moisture foods, RTE muskmelons, tomatoes, and alfalfa sprouts (de Moraes et al., 2017; Holden et al., 2024; Esteban-Cuesta et al., 2026; Jayeola et al., 2020). Additionally, several genes involved in the enterobacterial common antigen (ECA) biosynthesis showed a fitness effect in our TIS data. Specifically, several components of the *wec* gene cluster were identified to be relevant for the interaction of STM and SEN on both onions and alfalfa sprouts and have repeatedly been reported to contribute to fitness in *S. enterica* on various food matrices (de Moraes et al., 2017; Esteban-Cuesta et al., 2026; Holden et al., 2024; Jayeola et al., 2020). The fitness disadvantage of *wecF* STM mutants was confirmed in the competition experiments on onions and in LB, suggesting a nutrient-dependent fitness effect. Similarly, the RcsB-RcsC and the BarA-UvrY TCSs were relevant for SEN on both food matrices. The relevance of the RcsB-RcsC TCS may be due to its functions in regulating capsule synthesis (Stout and Gottesmann, 1990), activating virulence factors, or responding to osmotic and envelope stress (Davalos-Garcia et al., 2001). The BarA-UvrY TCS regulates the metabolic shift from glycolysis to gluconeogenesis in *E. coli* (Pernestig et al., 2003). This may support the hypothesis of an increased demand for de novo synthesis of certain nutrients by reutilizing intracellular resources. A linkage between this TCS and the expression of genes coding for secreted effectors has been observed in various gram-negative bacteria (Altier et al., 2000; Heeb and Haas, 2001; Herren et al., 2006; Pernestig et al., 2001). We note that in our data, the disruption of genes involved in secretion systems or the production of secreted effector proteins (*csgEF*, *invJG*, *prgI*, *ssaCDLPQV*, *spaP*) had a significant fitness effect, especially in SEN.

The acidic environment in onions seems to trigger various host-invasion mechanisms in SEN, perhaps because it reflects the pH drop during gastrointestinal tract passage as well as the acidic shift occurring in *Salmonella*-containing vacuoles (SCV) or in macrophages (Alpuche Aranda et al., 1992; Chakraborty et al., 2015). Intrinsic antimicrobial substances and the immune system of onions, as well as cold stress, may additionally induce the expression of virulence factors (Schikora et al., 2008; Shah et al., 2013, 2014; Shirron and Yaron, 2011). T3SSs were also shown to be crucial in many endophytic bacteria for plant colonization (Pinski et al., 2019; Schikora et al., 2008) and during the interaction with foods of plant origin such as alfalfa sprouts and tomatoes (Brankatschk et al., 2014; de Moraes et al., 2017; Holden et al., 2024), probably due to its role in modulating the plant immune response (Deslandes and Rivas, 2012; Guo et al., 2009; Pinski et al., 2019).

Mutants with insertions in flagellar assembly genes were positively selected on both food matrices. This is in line with previous findings showing a positive fitness effect of flagella mutants during the colonization of different species of *Medicago* plants with *S*. Typhimurium (Iniguez et al., 2007) and of muskmelons with SEN (Esteban-Cuesta et al., 2026). Since motility is energetically costly (Schavemaker and Lynch, 2022), downregulation of flagellar expression whenever motility is less beneficial improves efficiency and provides a competitive growth advantage due to the reduced energetic expenditure. Additionally, *S. enterica* downregulates its motility under cell envelope and oxidative stress (Spöring et al., 2018), such as an acidic environment.

Considering the natural antimicrobial substances present in onions (Kyung, 2012; Lanzotti et al., 2013; Santas et al., 2010; Sharma et al., 2018) it is not surprising that the mutants deficient for the multidrug efflux pump TolC were strongly negatively selected in the TIS analyses of both STM and SEN. A previous study showed that disruption of *acrA*, which is part of the well-studied AcrAB-TolC efflux complex, resulted in a significant fitness disadvantage for SEN on almonds (Li et al., 2020). TolC exports a wide range of exogenous and endogenous compounds, including antibiotics and bile compounds (Morona et al., 1983), and contributes to *S*. Choleraesuis survival under acidic stress (Lee et al., 2016). Taken together, these findings support the need for *S. enterica* to withstand acidic conditions and antimicrobial compounds on fresh onions, identifying related genes as potential targets for future food safety interventions.

The key regulator of the oxidative stress response, OxyR (Storz et al., 1990), exhibited a dynamic fitness effect in the transposon screen of both STM and SEN on alfalfa sprouts. OxyR is involved in the cellular response to reactive oxygen species (ROS), which are part of the immune system of plants (Jones and Dangl, 2006). During the transposon library screen, the initial negative selection of *oxyR* mutants shifted to positive for the remainder of the experiments, confirming the results previously observed on muskmelons (Esteban-Cuesta et al., 2026). The TIS data also revealed that the initial fitness disadvantage conferred by the *oxyR* mutation was much milder on alfalfa sprouts than on fresh diced onions or when no food matrix was provided. This suggests that *oxyR* is less critical for survival on sprouts than on onions and in standard matrix-free conditions. This could be because of the inherent antioxidants in alfalfa sprouts or an effect of the competing background microbiota. Due to the relevance of *oxyR* in the transposon data, individual *oxyR* mutants were included in competition assays against the wild-type. These mutants performed highly variably in the assays, resulting in a high standard deviation. This could be attributed to the dual functionality of OxyR that can switch from transcriptional repressor to transcriptional activator of a multitude of genes beyond oxidative stress.

Holden et al. (2024) identified key fitness factors for *S. enterica* growing at room temperature on alfalfa sprouts. Surprisingly, considering that our work measures genes required during storage without growth at 8 °C, several of their findings align with our study, including roles in LPS biogenesis, DNA housekeeping, and flagella biosynthesis. The differences between our study and theirs are of interest as they may indicate differences due to growth versus storage. Among these differences, Holden et al. (2024) highlights the importance of iron storage for the growth of biotic and abiotic surfaces, which were found on muskmelons (Esteban-Cuesta et al., 2026) and tomatoes (de Moraes et al., 2017) but which we do not observe during storage at 8 °C.

Our findings should be interpreted in light of certain limitations. First, a key limitation of most TIS studies is the high inoculation level required to preserve library complexity. However, our data were validated in competition assays where many, but not all, individual mutant phenotypes were confirmed against the wild-type. Observed discrepancies between TIS and competition assay results likely arise from methodological differences: the TIS analysis accounts for interactions of all mutants within the library, whereas competition assays assess the growth of individual deletion mutants relative to the wild-type. However, the presence of opposing selection patterns as observed in the KEGG analysis could indicate biological complexity, but also certain inconsistency, or noise in the data. An additional limitation of our experimental setup is the high amount of natural background microbiota on alfalfa sprouts, which affects nutrient availability for *S. enterica* libraries and contributes to greater variability between experiments. However, we deliberately aimed to provide real-world data by incorporating the naturally occurring microbiota, which plays a key role in shaping the prevalence and interaction dynamics of foodborne pathogens such as *S. enterica*.

## 5. Conclusion

This study offers novel insights into the genetic determinants of the fitness of *S. enterica* on fresh diced onions and alfalfa sprouts. Using transposon insertion sequencing and validation via competition assays, we identified both shared and matrix-specific survival mechanisms across serovars. In onions, virulence-associated systems such as T3SS and efflux pumps may become activated and important for growth. *Salmonella oxyR* mutants, which are typically severely impaired, recovered in sprouts but not on onions or in matrix-free growth conditions. Our findings deepen our understanding of how *S. enterica* adapts to distinct produce environments and highlight potential molecular targets for future food safety interventions.

## Supplementary Material

Table S2

Figure S1

Figure S2

Figure S3

Figure S4

Table S1

## Figures and Tables

**Table 1:** Genes with a significant fitness effect for *S*. Typhimurium 14028 and *S*. Enteritidis P125109 on fresh-diced onions grouped by function.

Metabolic function	Genes	STM	SEN	STM14 Locus
DNA repair, recombination, and replication	*holC*	−	n.s.	STM14_5372.J
	*holD*	−	n.s.	STM14_5475
	*rpoC*	n.s.	+/−	STM14_4991
	*ydeJ/cinA*	n.s.	−/+	STM14_1830
	*xseA*	−	n.s.	STM14_3077
RNA degradation	*deaD*	−	n.s.	STM14_3962
	*pnp*	−	n.s.	STM14_3964
	*vacB*	−	n.s.	STM14_5250
Transcriptional regulator	*envR*	n.s.	−/+	STM14_4087
	*pocR*	n.s.	−/+	STM14_2525
	*sinR*	n.s.	−/+	STM14_0358
	PTR	n.s.	−/+	STM14_0016
	PTR	n.s.	−/+	STM14_1527
	PTR	n.s.	−/+	STM14_1646
	*marT_2*	n.s.	−/+	STM14_0037
	*rcsB*	n.s.	−/+	STM14_2801
	*ydiP*	n.s.	−/+	STM14_1646
Ribosome biogenesis	*yjgA*	−	n.s.	STM14_5328
	***typA***	−	−	STM14_4822
tRNA biogenesis	*queA*	n.s.	−/+	STM14_1868
Chaperones and co-chaperones	*spy*	n.s.	−/+	STM14_1588
Transporter	*corA*	−	n.s.	STM14_4754
	***yshA (ompL)***	−/+	−/+	STM14_4829
	PCT	n.s.	−/+	STM14_0889.RJ
	*nanT*	n.s.	−/+	STM14_1295
	*secA*	+/−	n.s.	STM14_0162
Membrane transport and secretion system	*csgEF*	n.s.	−/+	STM14_1304/05
	PP	n.s.	−/+	STM14_0384; STM14_2808
	*sseCD*	n.s.	−/+	STM14_1696/97
	*ssrA*	n.s.	−/+	STM14_1687
	***tolC***	−	−	STM14_3859
	*ulaA_1*	n.s.	−/+	STM14_1035
	*ydiMN*	n.s.	−/+	STM14_1651/54
Signaling and cellular processes	*etfA*	n.s.	−/+	STM14_1003
	*nlp_1*	n.s.	−/+	STM14_2923
	PRR	n.s.	−/+	STM14_0405; STM14_1526
	SEN4290	n.s.	−/+	550,537.33. peg.4529
Glycan, lipopolysaccharides, O-antigen, and enterobacterial common antigen biosynthesis	***dcrB***	−	−	STM14_4308
	*wecB*	−	n.s.	STM14_4718
	*wecD*	−	n.s.	STM14_4722
	***wecE***	−	−	STM14_4723
	*wecF*	−	n.s.	STM14_4725
	*ldcA*	−	n.s.	STM14_2176
	POMP	+	n.s.	STM14_2773
	POMP	n.s.	−/+	STM14_1612
	*rfc*	n.s.	−/+	STM14_1616
	*wcaE*	n.s.	−/+	STM14_2606
Infection, virulence, and defense	*avrA*	n.s.	−/+	STM14_3462
	*finP*	n.s.	−/+	STM14_5592
	*hilC*	n.s.	−/+	STM14_3465
	*pipC*	n.s.	−/+	STM14_1236
	*phoN*	n.s.	−/+	STM14_5193
	*pliC*	n.s.	−/+	STM14_1505
	*rck_2*	n.s.	−/+	STM14_3661
	*rmbA*	n.s.	−/+	STM14_4526
	*sopBD*	n.s.	−/+	STM14_1237; STM14_3550
	*sseF*	n.s.	−/+	STM14_1700
	*psiA*	n.s.	+/−	STM14_5591
	PCWH	n.s.	−/+	STM14_2358
	Phage	n.s.	−/+	STM14_3172
	Phage	n.s.	−/+	STM14_2430
	Phage	n.s.	−/+	STM14_1192
T3SS	*invJAG*	n.s.	−/+	STM14_3491; STM14_3495; STM14_3497
	*prgI*	n.s.	−/+	STM14_3472
	*ssaCDLPQV*	n.s.	−/+	STM14_1689; STM14_1690; STM14_1708; STM14_1710; STM14_1713; STM14_1714
	*sipC*	n.s.	−/+	STM14_3483
	*spaP*	n.s.	−/+	STM14_3489
	*PCP*	n.s.	−/+	STM14_1706
Flagella	*fliA*	n.s.	+/−	STM14_2374
	*flgC*	n.s.	+/−	STM14_1346
	*flhD*	n.s.	+/−	STM14_2341
Chemotaxis	PSSR	n.s.	−/+	STM14_3799
	*spi4_A* (HP)	n.s.	−/+	STM14_5118
Fimbriae	*fimW*	n.s.	−/+	STM14_0644
	*pefB*	n.s.	−/+	STM14_5544
	*safA*	n.s.	−/+	STM14_0352
	*sthAB*	n.s.	−/+	STM14_5514; STM14_5515
	*stfA*	n.s.	−/+	STM14_0234
Antimicrobial resistance	*acrAB*	n.s.	−	STM14_0560; STM14_0559
	*fimZ*	n.s.	−/+	STM14_0641
	*ompC*	n.s.	−/+	STM14_1848
Carbohydrate metabolism	*fumB*	n.s.	−/+	STM14_0886
	*gmpA*	n.s.	−/+	STM14_0896
	*manA*	+/−	n.s.	STM14_1769
	*manL_2*	n.s.	−/+	STM14_5450
	PMD	n.s.	−/+	STM14_3723
Purine catabolism	*allD*	n.s.	−/+	STM14_0618
Amino acid metabolism	*aroC*	−	n.s.	STM14_2933
	*eutS*	n.s.	−/+	STM14_3028
	*gor*	n.s.	−/+	STM14_4327
	*gpmA*	n.s.	−/+	STM14_0896
	*gshB*	n.s.	−/+	STM14_3739
	*pphB*	n.s.	−/+	STM14_3509
	*nanM*	n.s.	−/+	STM14_1293
	*tdcB*	n.s.	−/+	STM14_3926
Envelope and membrane	*envE*	n.s.	−/+	STM14_1493
	POMLP	n.s.	−/+	STM14_1513
	POMP	n.s.	−/+	STM14_4874
	*ybhM*	n.s.	−/+	STM14_0938
Fatty acid metabolism	*acd-6*	n.s.	−/+	STM14_1005
Osmotic stress	*yciEF*	n.s.	−/+	STM14_2092; STM14_2093
	*ygaU*	n.s.	−/+	STM14_3371
Conjugation	*traY*	n.s.	−/+	STM14_5596
PTS System	PIMP	n.s.	−/+	STM14_1036
	*ulaA_1*	n.s.	−/+	STM14_1035
Uncharacterized genes	Hypothetical proteins	n.s.	+	550.537.33. peg.1053; STM14_0337; STM14_0357; STM14_1197; STM14_1289; STM14_1401; STM14_2418; STM14_3789; STM14_4535; STM14_4997; STM14_5381; STM14_0643
	Putative cytoplasmic proteins	n.s.	+	STM14_0025; STM14_2888; STM14_0359; STM14_3960; STM14_4519; STM14_4993; STM14_4996; STM14_5175; STM14_5190
	Putative inner membrane proteins	n.s.	+	STM14_0398; STM14_0979; STM14_1937; STM14_1975; STM14_2258; STM14_2638; STM14_2728; STM14_4449
	Putative periplasmic proteins	n.s.	+	STM14_0399; STM14_0517; STM14_0660
	*ydiV*	n.s.	−/+	STM14_1632
	*ydiL*	n.s.	−/+	STM14_1655
	PDR	n.s.	−/+	STM14_1970
	Pseudogene	n.s.	−/+	STM14_3788

Mutants listed met the criteria for significance, as defined in the materials and methods section Genes in **bold** showed a significant fitness effect in both serovars.

−, conditions for which a statistically significant negative fitness effect was observed.

+, conditions for which a statistically significant positive fitness effect was observed.

+/−, positive fitness effect at the first sampling time point, followed by a negative fitness effect later.

−/+, negative fitness effect at the first sampling time point, followed by a positive fitness effect later.

n.s., genes did not fulfill both criteria for significance in the respective library. HP: hypothetical protein, PTR: putative transcriptional regulator, PCT: putative cation transporter, PP: putative permease; PRR: putative response regulator; POMP: putative outer membrane protein; PIMP: putative inner membrane protein; PCWH: putative cell wall-associated hydrolase; PCP: putative cytoplasmic protein, PSSR: putative serine sensor receptor; PMD: putative mannitol dehydrogenase, POMLP: putative outer membrane lipoprotein; PDR: putative dipicolinate reductase; T3SS: type III secretion system.

**Table 2:** Genes with a significant fitness effect for *S*. Typhimurium 14028 and *S*. Enteritidis P125109 on alfalfa sprouts grouped by function.

Metabolic function	Genes	STM	SEN	STM14 locus
Reactive oxygen species (ROS) stress response	***oxyR***	−/+	−/+	STM14_4959
Chaperone	*dsbA_2*	n.s.	–	STM14_4806
Transporter	*corA*	–	n.s.	STM14_4754
Flagella	*fliCD*	n.s.	+/−	STM14_2378; STM14_2380
	*flgKL*	n.s.	+/−	STM14_1354; STM14_1355
	*motA*	n.s.	+/−	STM14_2338
Capsule, envelope and lipopolysaccharides	*ldcA*	–	n.s.	STM14_2176
	*rcsB*	n.s.	−/+	STM14_2801
	*rfaK*	n.s.	−/+	STM14_4475;
	*rfaJ*	n.s.	–	STM14_4478
	*wecE*	n.s.	–	STM14_4723
Carbohydrate metabolism	*eda*	–	n.s.	STM14_2289
Uncharacterized	HP	n.s.	−/+	STM14_2047
	HP	n.s.	+/−	STM14_2339

Mutants listed met the criteria for significance. Genes in **bold**: genes with a significant fitness effect in both serovars.

−, conditions for which a statistically significant negative fitness effect was observed.

+, conditions for which a statistically significant positive fitness effect was observed.

+/−, positive fitness effect at the first sampling time point, followed by a negative fitness effect later.

−/+, negative fitness effect at the first sampling time point, followed by a positive fitness effect later. n.s., genes did not fulfill both criteria for significance in the respective library.

HP: hypothetical protein.

**Table 3:** Significantly enriched KEGG pathways associated with the interaction of *S*. Typhimurium 14028 and *S*. Enteritidis P125109 with fresh diced onions and alfalfa sprouts at 8°C along with corresponding significantly enriched GO terms.

Enriched pathway (q-value < 0.01)	Matrix	Strain	No of genes	Genes	Associated GO-terms (classic fisher ≤ 0.01)
Flagellar assembly	Onions	SEN	28	*flgACDEFGIKL*, *flhABCD*, *fliACEFGHIJKMOPZ*, *motAB*	GO:0040011, GO:0044780, GO:004781, GO:0051674, GO:0071973
	Sprouts	SEN	32	*flgACDEFGHJKLN*, *flhABD*, *motAB*, *fliACDEFGHIJKLMOQRZ*	GO:0040011, GO:0044780, GO:0044781, GO:0051674, GO:0071973, GO:0071978, GO:0097588
Two-component system	Onions	SEN	34	*barA*, bcfB*, *cheAR*, dcuB*, envZ*, fimZ*, *flhCD*, *fliAC*, iroN*, ***lpfB***, *motA*, *narY*, ompR*, ***pagD***, pagO*, pgtEP*, ***phoN***, *phoQ*, ***qseC***, rcsAB*, ***ssrA***, ***sthAB***, ***tolC***, ***tsr_3***, *uvrY*, ***wecC***, ygiK*, STM14_3842*	
	Sprouts	SEN	19	*fliAC*, *cheAMRWY*, *motA*, *flhD*, *uvrY*, rcsABC*, barA*, ***tolC***, envZ*, ompR*, ***wecBC***	GO:0000160
*Salmonella* infection	Onions	SEN	17	***avrA***, spvCD*, *fliC*, *htpG*, pipB2*, ***prgIJ***, ***sipAC***, ***sopB***, sopD-2*, ***sseFI***, sseL*, ***STM14_4996***	
Bacterial chemotaxis	Sprouts	SEN	9	*cheAMRWY*, *motAB*, *fliGM*	GO:0006935
RNA degradation	Onions	STM	5	***deaD***, ***pcnB***, ppk*, ***pnp***, ***vacB***	
Mismatch repair	Onions	STM	5	***xseA***, ***dam***, ***uvrD***, ***holCD***	GO:0009314
Biosynthesis of nucleotide sugars	Sprouts	STM	4	*flmB*, ***wecBCE***	GO:0009246

Gene lists were generated based on a *p*_adj_ < 0.05 in at least one *matrix*/*no matrix* or inoculum/time point comparison. **Bold** genes indicate negative selection between sampling time points. Underlined genes indicate positive selection between time points. Genes marked with “*” exhibited a shift in selection over the incubation period, changing from negative to positive selection or vice versa.

**Table 4:** Competition assays on fresh diced onions and alfalfa sprouts at 8 °C.

Gene	Matrix	Locus Tag	Function	TIS		Competition assay														
						Matrix					LB					PBS				
				STM	SEN	CI_as_	stdev_as_	CI_s_	stdev_s_	Outcome	CI_as_	stdev_as_	CI_s_	stdev_s_	Outcome	CI_as_	stdev_as_	CI_s_	stdev_s_	Outcome
*corA*	Onions	STM14_4754	Magnesium. nickel. cobalt transporter CorA	–	n.s	0.67	0.53	0.90	0.66	○	0.16	0.06	0.16	0.03	↓	0.85	0.02	0.93	0.32	↙
	Sprouts					0.63	0.22	0.42	0.25	↓										
*deaD*	Onions	STM1_3962	ATP-dependent RNA helicase DeaD	–	n.s.	1.28	1.22	1.01	0.43	○	0.22	0.20	0.12	0.04	↓	0.89	0.12	0.89	0.35	○
*holC*	Onions	STM14_5372	DNA polymerase III subunit chi	–	n.s.	x	x	0.17	0.11	↓	x	x	0.02	0.02	↓	x	x	1.05	0.12	○
*holD*	Onions	STM14_5475	DNA polymerase III subunit psi	–	n.s.	x	x	0.62	0.01	↓	x	x	0.01	0.00	↓	x	x	0.90	0.43	○
*ldcA*	Onions	STM14_2176	L. D-carboxypeptidase A	–	n.s.	0.03	0.03	0.10	0.10	↓	0.31	0.13	0.60	0.25	↓	0.19	0.11	0.25	0.15	↓
	Sprouts					0.27	0.33	0.16	0.13	↓										
*oxyR*	Sprouts	STM14_4959	DNA-binding transcriptional regulator OxyR	–/+		17.32	13.42	2.66	1.73	↖	0.06	0.08	0.03	0.02	↓	/	/	/	/	/
*pnp*	Onions	STM14_3964	Polynucleotide phosphorylase/polyadenylase	–	n.s.	0.34	0.16	0.56	0.23	↓	0.12	0.07	0.15	0.09	↓	/	/	/	/	/
*tolC*	Onions	STM14_3859	Outer membrane channel protein	–		x	x	0.05	0.05	↓	x	x	0.41	0.20	↓	x	x	1.10	0.49	○
*typA*	Onions	STM14_4822	GTP-binding protein	–		0.71	0.23	0.46	0.10	↓	0.05	0.03	0.06	0.02	↓	/	/	/	/	/
*vacB*	Onions	STM14_5250	Exoribonuclease R	–	n.s.	2.96	2.89	2.22	2.07	○	0.38	0.42	0.11	0.11	↓	/	/	/	/	/
*wecC*	Onions	STM14_4719	UDP-*N*-acetyl-D-mannosamine dehydrogenase	(–)	n.s.	1.15	0.21	0.77	0.08	↘	0.79	0.42	4.41	6.64	○	/	/	/	/	/
*wecD*	Onions	STM14_4722	TDP-fucosamine acetyltransferase	–	n.s.	1.30	1.38	1.33	0.78	○	0.33	0.51	0.08	0.10	↓	/	/	/	/	/
*wecE*	Onions	STM14_4723	TDP-4-oxo-6-deoxy-d-glucose transaminase	–		0.61	0.49	x	x	○	0.39	0.32	x	x	↓	/	/	/	/	/
*wecF*	Onions	STM14_4725	4-Alpha-L-fucosyltransferase	–	n.s.	0.04	0.03	0.06	0.05	↓	0.29	0.13	0.27	0.09	↓	/	/	/	/	/
*xseA*	Onions	STM14_3077	Exodeoxyribonuclease VII large subunit	–	n.s.	0.09	0.08	0.82	0.31	↙	0.09	0.16	2.65	3.77	↙	0.64	0.19	1.68	1.28	↙
	Sprouts			(–)		0.34	0.15	1.04	0.23	↙										
*yjgA*	Onions	STM14_5328	ribosome associated protein	–	n.s.	x	x	0.17	0.07	↓	x	x	0.06	0.02	↓	x	x	0.97	0.04	○

All genes were tested for the time point I-d_5_.

TIS: Transposon insertion sequencing. −: mutation resulted in a negative fitness effect in the TIS analysis, +: mutation resulted in a positive fitness effect in the TIS analysis.

(): mutation fulfilled only one criterium for significance, n.s.: mutation did not fulfill both criteria for significance.

CI: competitive index = timepoint (mutant/wild-type) / inoculum (mutant/wild-type), average of at least two independent experiments, S: sense – single-gene deletion mutant with a kanamycin resistance cassette inserted in the sense orientation of the gene; AS: antisense – single-gene deletion mutant with a chloramphenicol resistance cassette inserted in the antisense orientation of the gene. ↓ = single-gene deletion mutants with a significant growth disadvantage in competition assays compared to wild-type (CI + stdev < 1); ↑ = advantage in competition assays compared to wild-type (CI – stdev > 1); ○ = no effect, ↙ negative effect in antisense mutant only, ↖ positive effect in antisense mutant only, ↘ negative effect in sense mutant only. LB: Luria-Bertani broth; PBS: phosphate-buffered saline; x: SGD not available; in brackets (): fitness effects which did not fulfill the criteria for significance; /: Competition assay was not performed.

## Data Availability

The data are provided in the supplementary material.

## Appendix A. Supplementary data

Supplementary data to this article can be found online at https://doi.org/10.1016/j.ijfoodmicro.2026.111696.
