# Supplementary material for: A screen of Salmonella enterica mutants interacting with fresh onions and alfalfa sprouts
Source: Int J Food Microbiol. Author manuscript; Available in PMC 2026 May 16. (PMC13152249; doi:10.1016/j.ijfoodmicro.2026.111696)
Supplement: Figure S1 [file NIHMS2165707-supplement-Figure_S1.pdf]

A

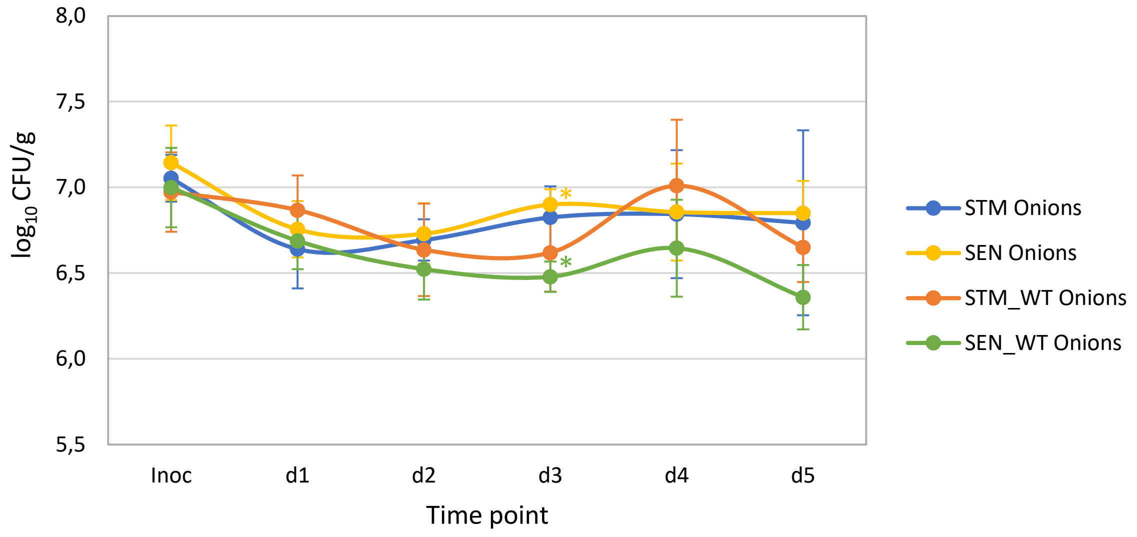

B

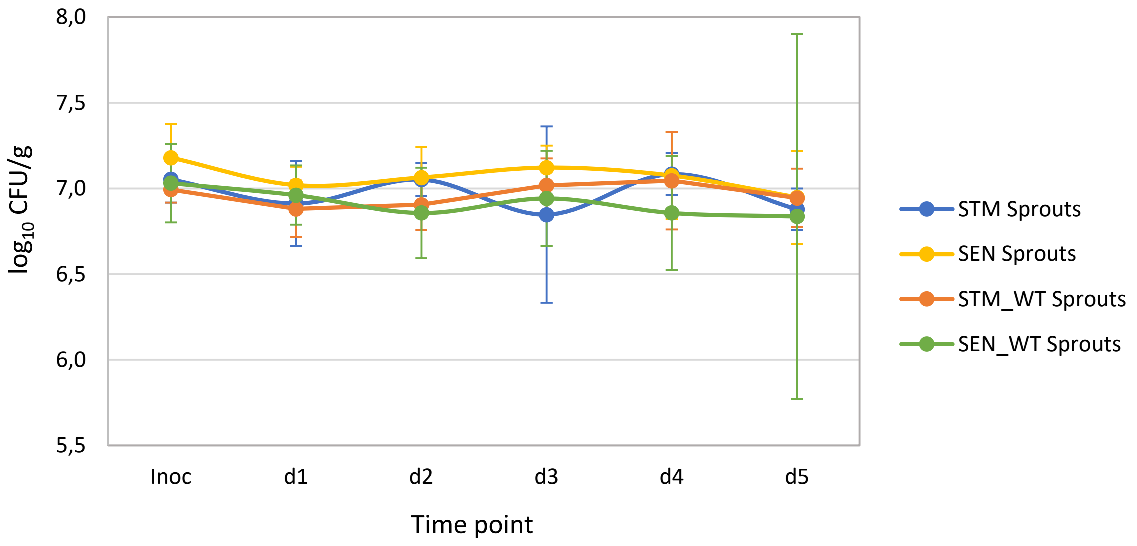

Figure S1: Population dynamics of the transposon insertion sequencing libraries in comparison to the wild type. Dynamics of the barcoded transposon libraries on fresh diced onions (**A**) and alfalfa sprouts (**B**) at 8 °C were quantitatively assessed in comparison to the wild type (WT). Sampling was carried out at the following time points: Inoculum (Inoc), 1 h after incubation (d_1), continuing every day for five days (d_2-d_5). Data represent the average of five biological replicates. Error bars represent the standard deviation. *: statistically significant difference between the library and its respective wild type. STM: *S.* Typhimurium 14028, SEN: *S.* Enteritidis P125109.
