# Supplementary material for: A screen of Salmonella enterica mutants interacting with fresh onions and alfalfa sprouts
Source: Int J Food Microbiol. Author manuscript; Available in PMC 2026 May 16. (PMC13152249; doi:10.1016/j.ijfoodmicro.2026.111696)
Supplement: Figure S2 [file NIHMS2165707-supplement-Figure_S2.pdf]

A

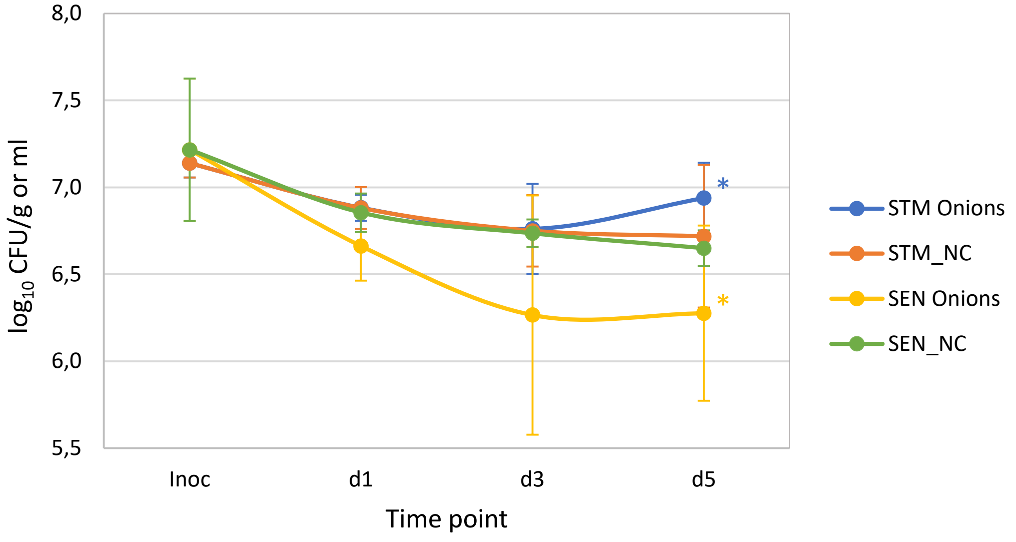

B

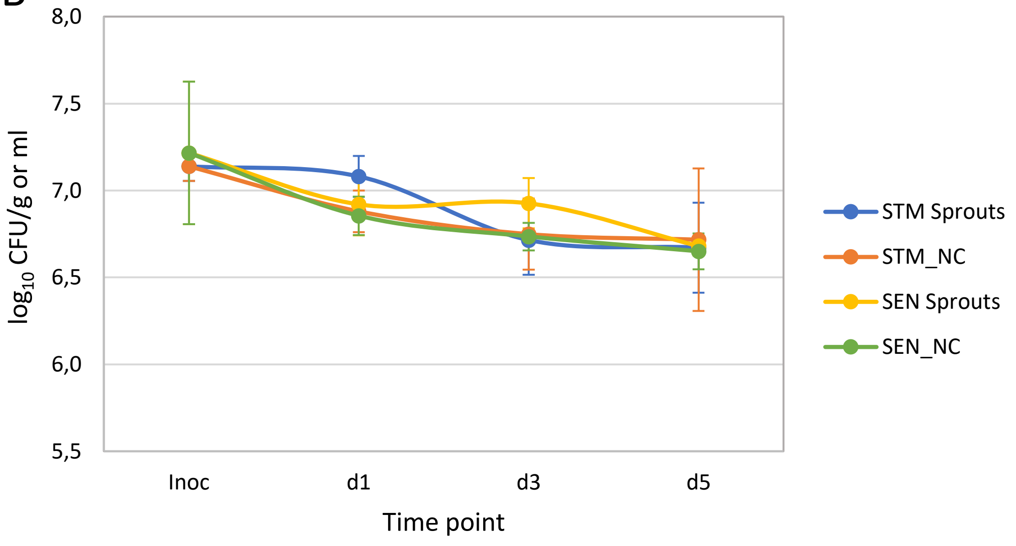

Figure S2: Dynamics of the transposon insertion sequencing libraries during the screenings at 8 °C. Population dynamics of the transposon insertion sequencing libraries on fresh diced onions (**A**), alfalfa sprouts (**B**), and in PBS (NC). Sampling timepoints: Inoculum (Inoc), 1 h after incubation (d_1), 48h after incubation (d_3) and 96h after incubation (d_5).

Data represent the average of five biological replicates. Error bars represent the standard deviation. NC: PBS-control. *: statistically significant difference between the TIS libraries. STM: *S*. Typhimurium 14028. SEN: *S*. Enteritidis P125109
