# Supplementary material for: A screen of Salmonella enterica mutants interacting with fresh onions and alfalfa sprouts
Source: Int J Food Microbiol. Author manuscript; Available in PMC 2026 May 16. (PMC13152249; doi:10.1016/j.ijfoodmicro.2026.111696)
Supplement: Figure S3 [file NIHMS2165707-supplement-Figure_S3.pdf]

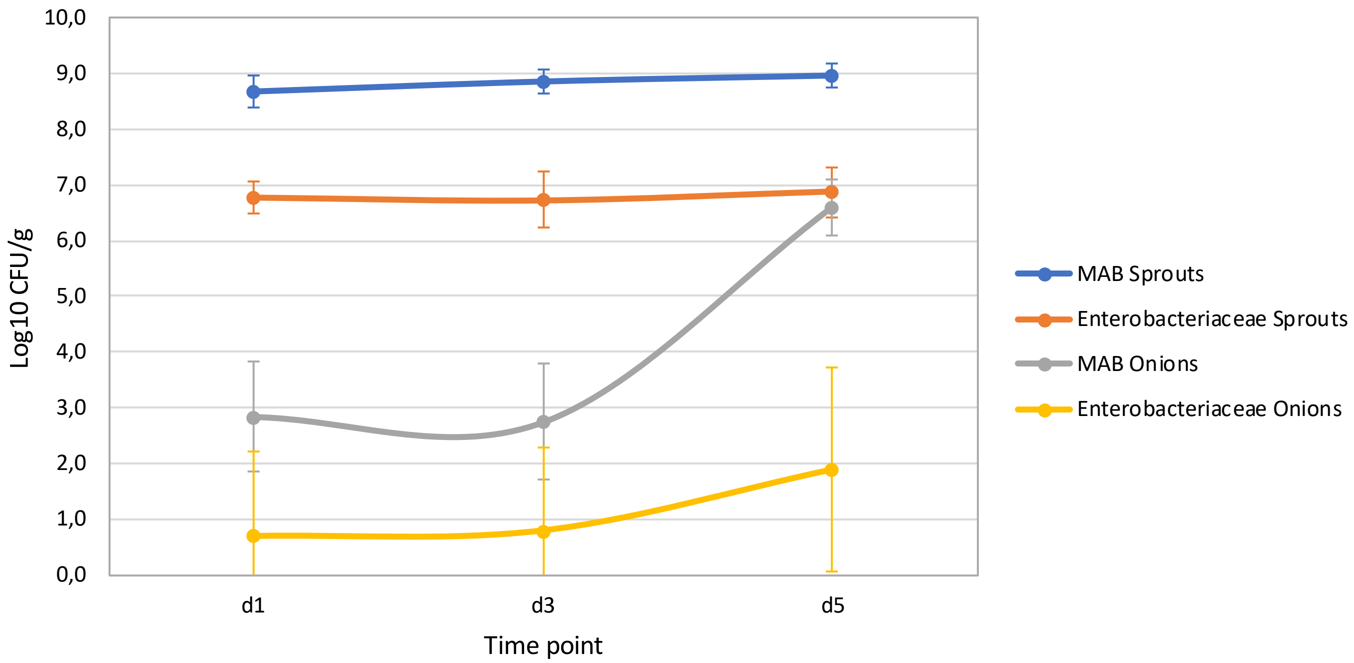

Figure S3. Background microbiota on diced onions and alfalfa sprouts during screenings of *S. enterica* TIS libraries at 8 °C Mesophilic aerobic bacteria (MAB) and *Enterobacteriaceae*, were quantitative assessed during the screenings of the TIS libraries at 8 °C. Sampling time points: one hour after incubation (d_1), 48 h after incubation (d_3), 96 h after incubation (d_5). Data represent averages from five biological replicates. Error bars represent the standard deviation.
