# Supplementary material for: A screen of Salmonella enterica mutants interacting with fresh onions and alfalfa sprouts
Source: Int J Food Microbiol. Author manuscript; Available in PMC 2026 May 16. (PMC13152249; doi:10.1016/j.ijfoodmicro.2026.111696)
Supplement: Figure S4 [file NIHMS2165707-supplement-Figure_S4.pdf]

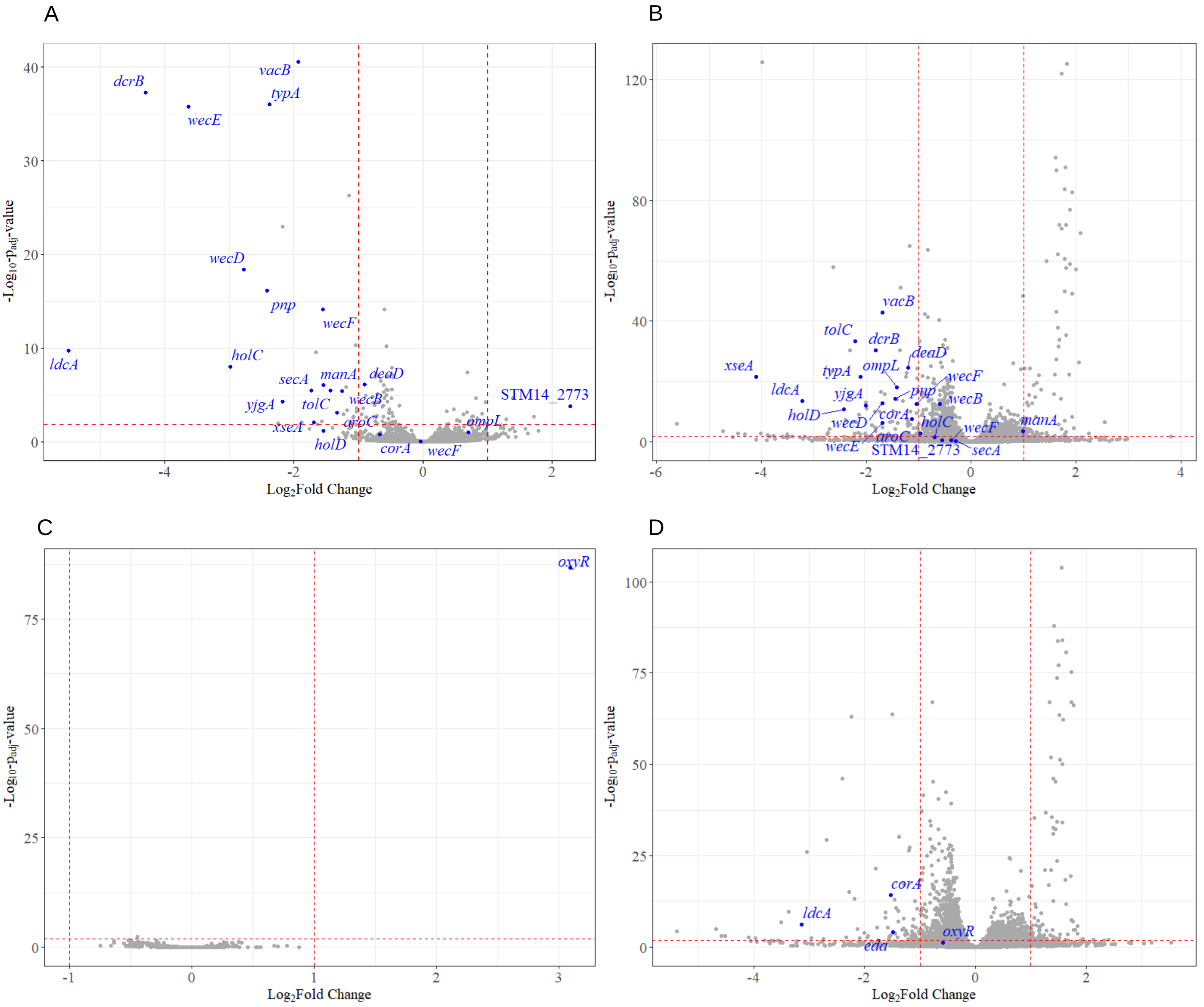

Figure S4a. Changes in the mutant abundances of *S.* Typhimurium 14028 insertion mutants during interaction with diced onions (A, B) and alfalfa sprouts (C, D) at 8°C. The frequency of STM insertion mutants on onions was compared between A. 1 h after incubation and 96 h of storage (d$_1$-d$_5$) and B. inoculum and 48 h of storage (I-d$_3$); on alfalfa sprouts the same timepoints were compared, C: d$_1$-d$_5$ and D: I-d$_3$. Mutations in genes that fulfilled both criteria for significance during the transposon screen are labeled in blue; insertions in intergenic regions are not included in the figures.

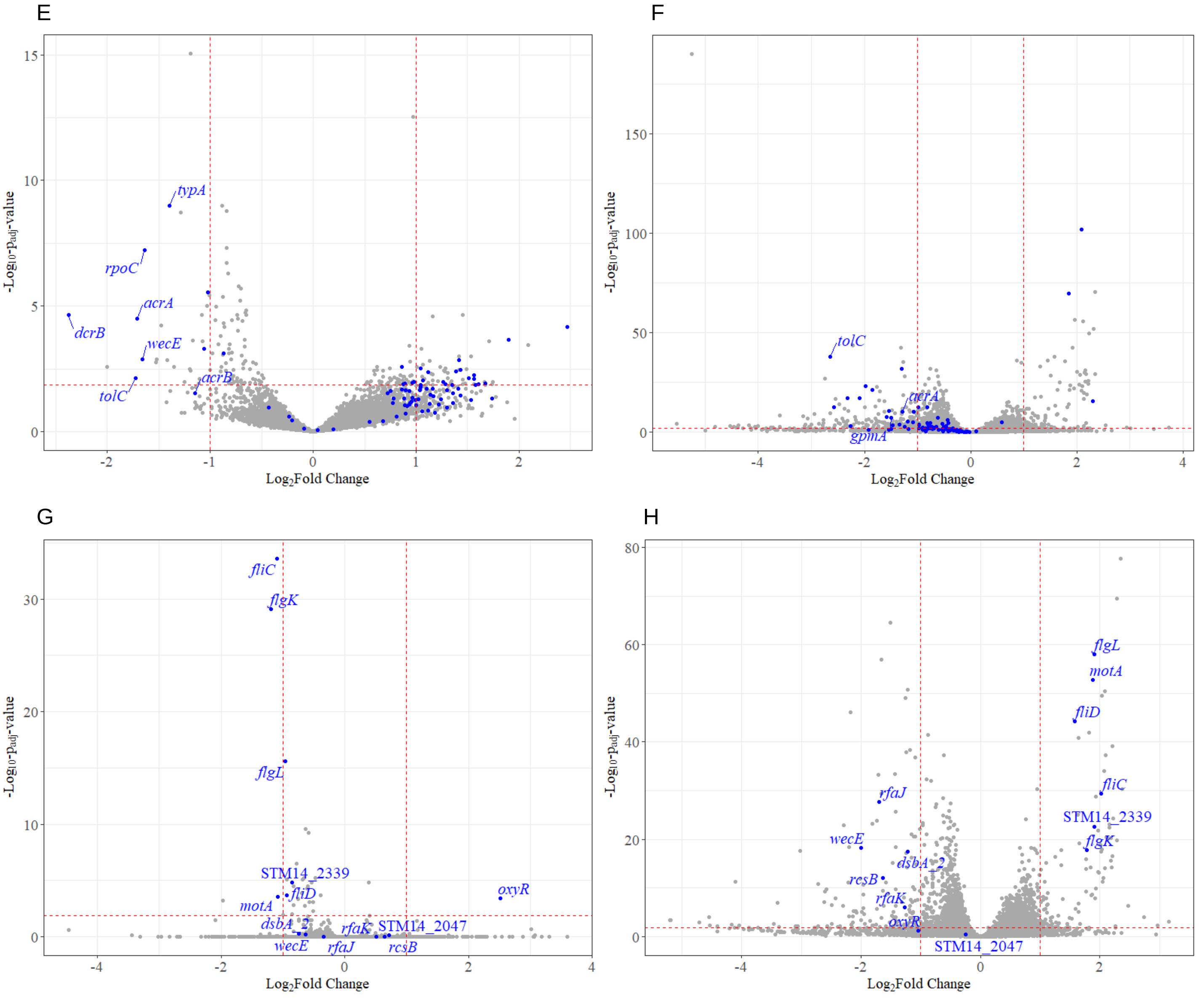

Figure S4b. **Changes in the mutant abundances of S. Enteritidis P125109 insertion mutants during interaction with diced onions (E, F) and alfalfa sprouts (G, H) at 8°C.** The frequency of SEN insertion mutants on onions was compared between A. 1 h after incubation and 96 h of storage (I-d_1), B. inoculum and 48 h of storage (I-d_3). On alfalfa sprouts the same timepoints were compared, G: d_1-d_5 and H: I-d_3.

In blue: mutations in genes that fulfilled both criteria for significance during the transposon screen; for SEN on onions only mutations that showed a $log_2fc \geq 2.5$ between timepoints are labeled, insertions in intergenic regions are not included in the figures
